# Macrodamage Accumulation Model for a Human Femur

**DOI:** 10.1155/2017/4539178

**Published:** 2017-08-29

**Authors:** Farah Hamandi, Tarun Goswami

**Affiliations:** ^1^Department of Biomedical, Industrial and Human Factors Engineering, Wright State University, Dayton, OH 45435, USA; ^2^Mechanical Engineering and Economic Sciences, Institute for Materials Science and Welding, Graz University of Technology, Kopernikusgasse 24/I, 8010 Graz, Austria; ^3^Department of Orthopedic Surgery, Sports Medicine and Rehabilitation, Miami Valley Hospital, Dayton, OH 45409, USA

## Abstract

The objective of this study was to more fully understand the mechanical behavior of bone tissue that is important to find an alternative material to be used as an implant and to develop an accurate model to predict the fracture of the bone. Predicting and preventing bone failure is an important area in orthopaedics. In this paper, the macrodamage accumulation models in the bone tissue have been investigated. Phenomenological models for bone damage have been discussed in detail. In addition, 3D finite element model of the femur prepared from imaging data with both cortical and trabecular structures is delineated using MIMICS and ANSYS® and simulated as a composite structure. The damage accumulation occurring during cyclic loading was analyzed for fatigue scenario. We found that the damage accumulates sooner in the multiaxial than in the uniaxial loading condition for the same number of cycles, and the failure starts in the cortical bone. The damage accumulation behavior seems to follow a three-stage growth: a primary phase, a secondary phase of damage growth marked by linear damage growth, and a tertiary phase that leads to failure. Finally, the stiffness of the composite bone comprising the cortical and trabecular bone was significantly different as expected.

## 1. Introduction

**Figure 1:**
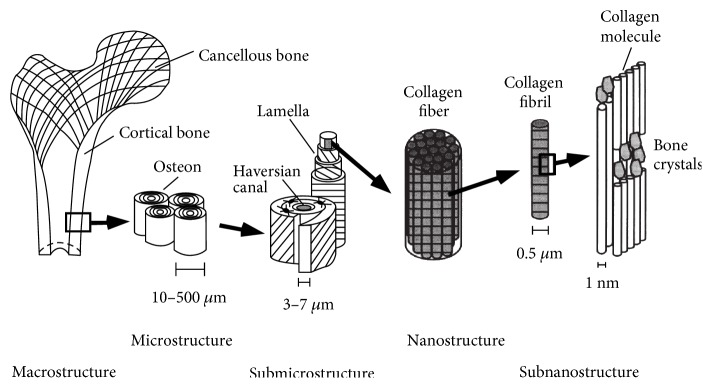
Hierarchal structure of the bone [2].

In order to understand the bone fracture, it is very important to study the macrodamage of the bone with respect to mechanical and physiological loads. Bone tissue is a complex, multiphasic, heterogeneous, anisotropic, and highly hierarchized material structure. Predicting and preventing bone fracture is a very important area in orthopaedics given the volume of fractures that occurs annually. From a macroscopic point of view, bone tissue is divided into two types: the trabecular bone with 50–95% porosity [1] and the cortical bone with 5–10% porosity [1]. Bone tissue can be divided into five levels [2], which are macro, meso, micros, submicro, and nanostructure. The macrostructure level is the whole bone, which ranges from several millimeters to several centimeters, as shown in Figure 1. In this paper, an attempt has been made to establish a detailed understanding of the bone tissue mechanical behavior as it is important in the device design and to derive implant life. Correspondingly, an accurate damage prediction model for a bone tissue is needed in order to predict the fracture of the bone or the reliability of a bone-implant structure.

Numerous damage models were proposed using the macrostructure of the bone. However, each model has made an assumption regarding the mechanical properties, loading conditions, or the structure of the bone. These assumptions have not given realistic predictions for the damage accumulation in a bone. Depending on the mechanical properties of bone tissue, bone damage models can be divided into elastic-viscoplastic, elastoplastic, and plastic damage models. In addition, depending on the damage type, bone damage models can be divided into electromagnetic, fracture, bending, and fatigue damage models. The elastic-viscoplastic damage models take into consideration that the bone has elastic, plastic, and viscus material properties.

Recently, several models have been proposed that describe the damage model of the bone as an elastic viscoplastic model such as Keyak and Rossi [3]. They proposed fracture load by using finite element models and several failure theories [3]. However, they used isotropic material properties for bone tissue. Some studies proposed elastoplastic damage modes as well. These models take into account elastic and plastic material properties such as in the Garcia et al. study [4] and the Fondrk et al. study [5]. They proposed elastic plastic damage models for bone tissue and developed a model for cortical bone tissue only. Other studies proposed plastic damage models, which take into consideration that the bone has plastic material properties only. In addition to the mechanical properties, the loading conditions have a significant effect on the macrodamage accumulation of the bone. Some studies analyzed only tension, compression, or three-point bending [6].

**Figure 2:**
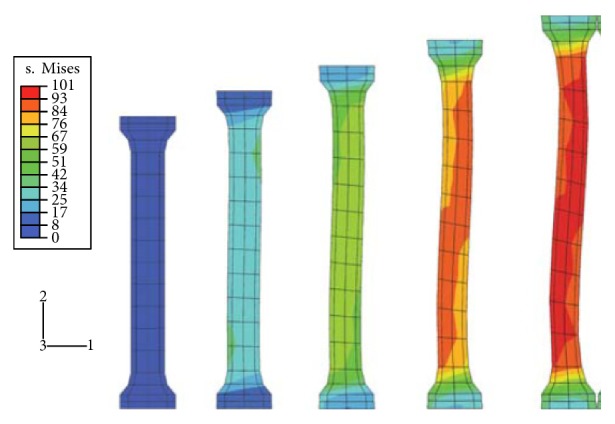
Garcia et al.'s [8] elastic-viscoplastic damage model finite element analysis on a cortical bone specimen.

**Figure 3:**
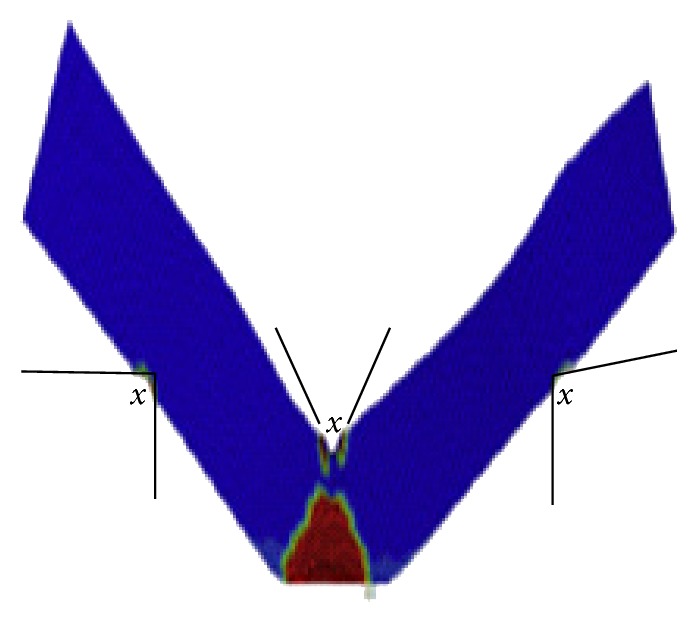
Ridha and Thurner's [9] finite element model.

**Figure 4:**
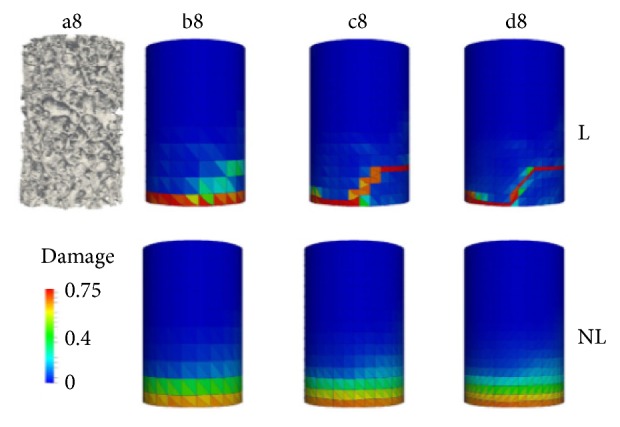
Hosseini et al.'s [12] plastic damage model for the trabecular bone.

**Figure 5:**
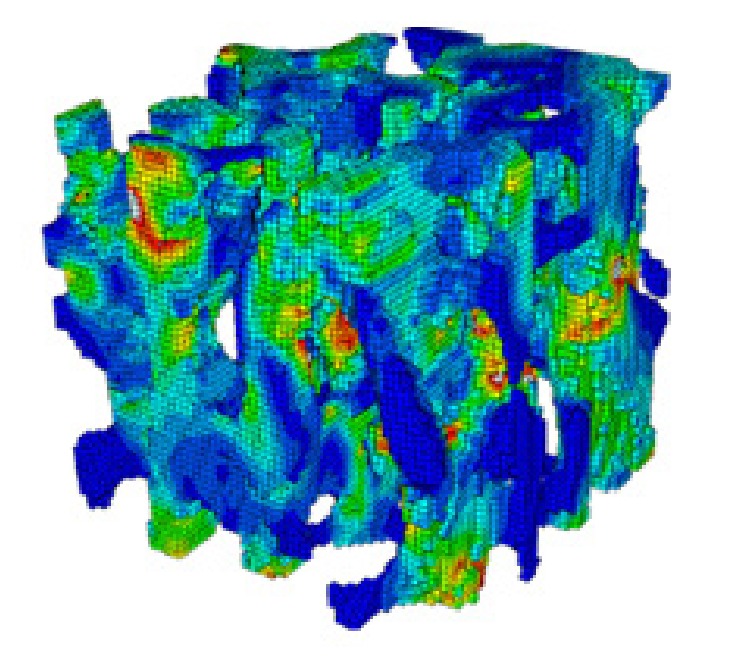
Hambli's [13] fatigue damage model.

Zlámal et al. proposed a numerical model for trabecular tissue using compression test and time-lapse X-ray radiography and three-point bending test of single trabecula [6]. Besides all of that, the main challenge that has been faced was to design a model for the bone that contains together the cortical and trabecular components of the bone. Some studies have worked only on the cortical component, such as Natali et al. [7]. Figures 2 and 3 show the use of a small sample from the femur to perform the finite element simulations. Other studies assumed that the damage starts at the trabecular components, so they created the damage models for the trabecular bone only, such as Charlebois et al. [10], Hambli [11], and Hosseini et al. [12]. Figures 4 and 5 show the use of a micro-CT to create small samples to perform the finite element simulations. In this paper, an attempt has been made to create a 3D model of the femoral bone that considers the anisotropic material properties of bone tissue and loads from realistic gait cycle to understand how damage accumulates in human bone tissue.

## 2. Material and Methods

### 2.1. Finite Element Modeling

Because of the difficulty in studying the macrodamage accumulation of the bone in vivo, mathematical and phenomenological models were used to simulate physiological conditions. A three-dimensional model of the femoral bone was created. Hip fractures are currently treated by trauma instrumentation. The choice of the biomaterial constituting the prosthesis determines the reliability. Hence, failure predictions in bone and bone-implant stability must be thoroughly investigated on computational models.

#### 2.1.1. Creating the Model

**Figure 6:**
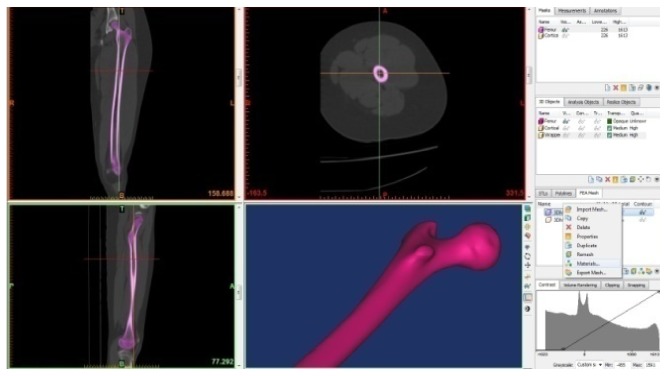
Creating the 3D model of the femoral bone using MIMICS.

**Figure 7:**
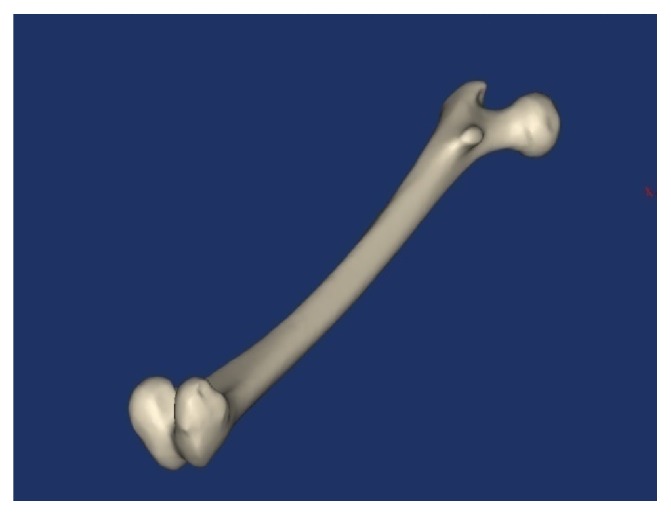
Femoral bone model.

A femur bone model was developed in three steps. Firstly, CT images for the femur were taken from a normal healthy femoral bone. Secondly, the CT images have been imported into the MIMICS 13.0 program to create a 3D model of the femoral bone, as shown in Figure 6. The cortical and trabecular components were created depending on the difference in density between them. Thirdly, the final model has been imported into SolidWorks (Dassault Systèmes SolidWorks Corp., Concord, MA, USA) to make the final improvements. The final model of the femoral bone that has both the cortical and the trabecular parts is shown in Figure 7.

#### 2.1.2. Material Definition

The proposed material properties of the bone consider the anisotropic and nonhomogeneity of the bone with its two types, the cortical and the trabecular. The trabecular bone is a spongy region; its density is lower than that of the cortical region, which is the hard and dense part of the bone. There are various procedures that have been performed to approximate the modulus of elasticity (*E*) of the bone depending on Hounsfield units (HU) and density (*ρ*) [14–16]. To give a realistic approximation for the bone tissue material properties, nine elastic constants must be provided depending on the orientation of the principal axes of orthotropy. While it is straightforward to assign the principal axes to the cortical zone, it is very challenging for the trabecular zone. In this study, both the cortical and trabecular zones have been divided into eight smaller segments. Then, each segment has been divided into ten material groups.

**Figure 8:**
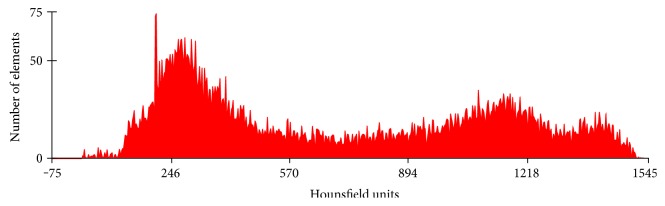
Hounsfield unit (HU) distribution across the femoral bone CT images.

Within the MIMICS program, the Hounsfield units (HU) were used to calculate the density (*ρ*) across each segment, and then the young's modulus (*E*) has been calculated in the radial, axial, and circumferential directions.  Figure 8 shows the HU distribution across the femoral bone CT images.

**Figure 9:**
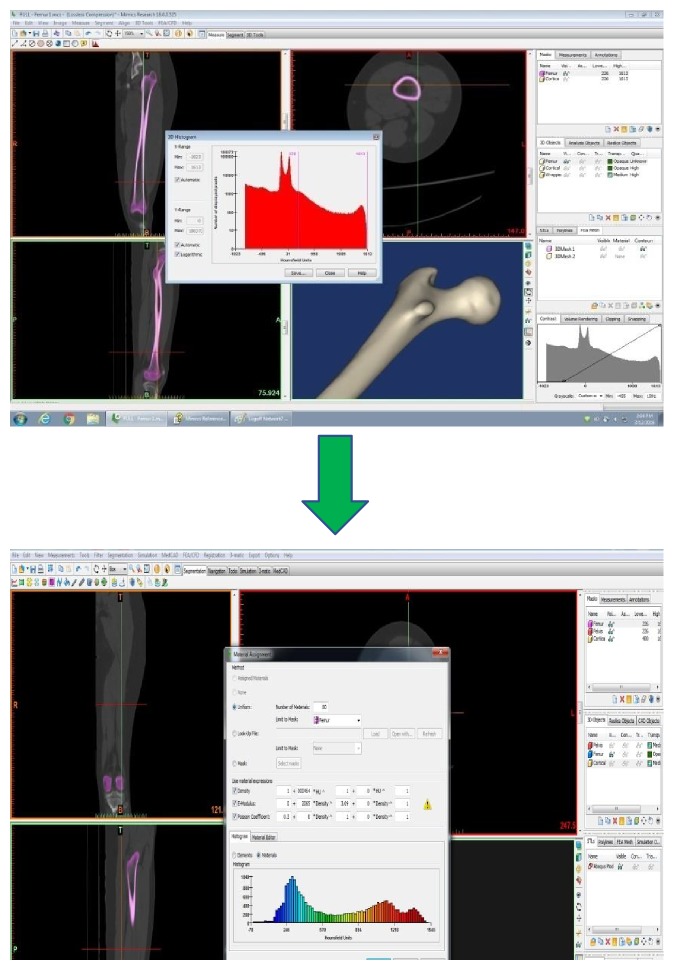
Material definition: the femoral bone model was imported in MIMICS, and different material properties were assigned by relating the bone mineral density with Hounsfield units. The colors have been modified to be green-blue colors for the trabecular material groups and yellow-red colors for the cortical material groups.

The mathematical relationship between Hounsfield units (HU) and effective density (*ρ*) that has been applied in MIMICS is as follows:

(1) $$\begin{array}{c} \rho =0.0000464HU+1, \end{array}$$

where the unit for the effective density (*ρ*) is g/cm^3^. The CT slices were used to align the orientation of each segment material. Also, the orthotropic relationships between the elastic constants and the density are different for the cortical and trabecular parts as described earlier, as shown in Table 1. Also, Figure 9 shows the procedure that has been used to find the material properties for each part of the femoral bone that has been used in this study.  Table 2 shows the 80 material groups with their densities and the nine elastic constants. The colors have been modified to be green-blue colors for the trabecular material groups and yellow-red colors for the cortical material groups, so one can differentiate between them, as shown in the last step. Finally, the material properties have been imported into ANSYS Workbench 16.2 (ANSYS Inc., Canonsburg, PA, USA). This procedure has been discussed in detail in the literature [14–16]. Additionally, to validate the importance of studying the bone as a composite material, the finite element simulation for each part of the bone has been done separately also. These simulations are extremely important in order to understand the effect of each zone on the whole bone.

#### 2.1.3. Finite Element Mesh

**Figure 10:**
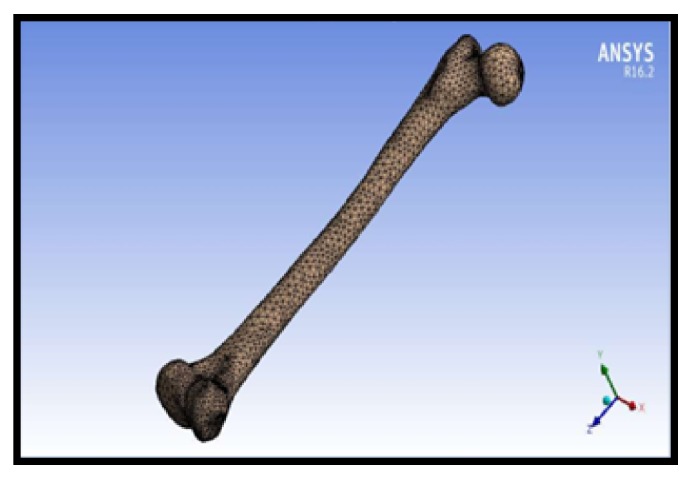
Meshing with tetrahedral elements.

Tetrahedral element type was used in this study with a minimum element size of 0.02 mm, as shown in Figure 10. The final model, which has been modified by SolidWorks, was imported into ANSYS Workbench 16.2. The finite element mesh was adapted automatically through the program. Following mesh convergence checks, the total number of elements was 26,898 for the whole femur, 22,328 elements in the cortical bone, and 4570 in the trabecular bone. In order to achieve repetitions of results within five percent, the meshing was refined with small increment size.

#### 2.1.4. Loads and Boundary Conditions

**Figure 11:**
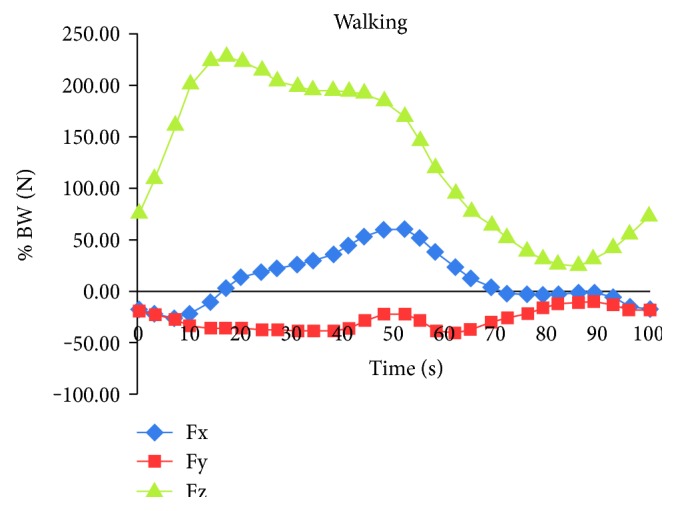
The force components of the hip contact force for walking [17].

To mimic physiological loading during normal walking, the reconstructed gait loads in the model were applied as a time-dependent analysis along its longitudinal axis. The gait cycle for walking was imported from the HIP98® program. In this program, total hip replacement joints on different patients were studied, and their movements were compared with the normal movements during different activities [17]. For the uniaxial loading, the equivalent maximum stress from the gait cycle was converted into a single load cycle. For the multiaxial loading condition from the gait pattern during walking, the initial applied triaxial load (Fz = vertical direction force, Fy and Fx = anterior–posterior and medial–lateral forces, resp.). A model fully fixed at the distal end was used in this study. The body weight acted on the femoral head and muscular force acted on the proximal femur. The hip contact, which transfers load from the upper body to the lower limbs, was investigated under static and dynamic conditions. The dynamic loads of the hip contact are shown in Figure 11 for walking condition. In this study, 10^6^ numbers of cycles have been used assuming that the average number of human walking cycles in one year is 1,000,000/year.

### 2.2. Phenomenological Bone Macrodamage Model

Goswami investigated phenomenological life prediction methods in great detail [18–21]. The macroscopic deformable bodies can be described via continuum mechanics. The main assumption made considers the nonhomogeneous anisotropic material properties of the bone tissue for both the cortical and trabecular bones. Since we assumed our model to be a composite material with different Young's moduli in the cortical and trabecular zones, an assumption was made that the strain in the cortical and trabecular zones is the same. Thus, we invoked a strain-based concept in damage modeling. The main material properties that are considered in creating the macrodamage accumulation model of the bone tissue are modulus of elasticity, fatigue strength coefficient, fatigue ductility coefficient, fatigue strength exponent, and fatigue ductility exponent. The first assumption in creating the model is to consider the elastic and plastic components as follows:

(2) $$\begin{array}{c} \varepsilon_{t}=\varepsilon_{e}+\varepsilon_{p}, \end{array}$$

where *ε*_t_ represents the total strain, *ε*_e_ the elastic strain, and *ε*_p_ the plastic strain. According to Hook's law,

(3) $$\begin{array}{c} \sigma =E\varepsilon , \end{array}$$

where *E* denotes the modulus of elasticity and *σ* the total stress. As it is important to take the nonhomogeneity of the bone tissue, the total strain can be expressed as follows:

(4) $$\begin{array}{c} \varepsilon_{kl}=\varepsilon_{kl\left(Elastic\right)}+\varepsilon_{kl\left(Plastic\right)}, \end{array}$$

where *ε*_kl_ represents the strain into different directions.

**Figure 12:**
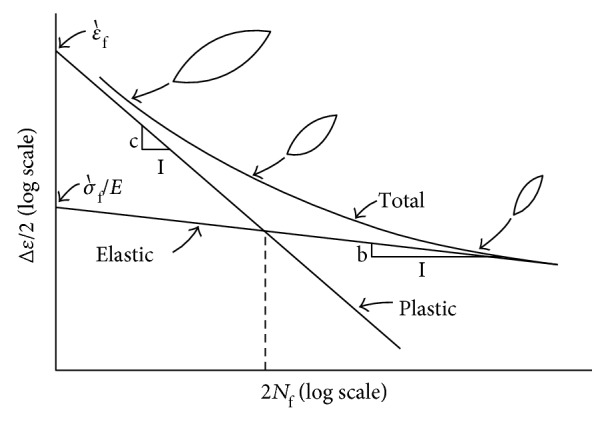
Strain-life curve [22].

According to the Coffin-Manson relation for the strain-life curve that is shown in Figure 12, the elastic and plastic parts can be expressed as follows:

(5) $$\begin{array}{c} \varepsilon_{kl}=\frac{{\overset{`}{\sigma }}_{f}}{C_{ijkl}}{\left(2N_{f}\right)}^{b}+{\overset{`}{\varepsilon }}_{f}{\left(2N_{f}\right)}^{c}, \end{array}$$

where ${\overset{`}{\sigma }}_{f}$ is the fatigue strength coefficient, ${\overset{`}{\varepsilon }}_{f}$ is the fatigue ductility coefficient, *b* is the fatigue strength exponent, *c* is the fatigue ductility exponent, and **C**_ijkl_ are the elasticity tensor components. There are three types of fluctuating stresses. These are fully reversed, repeated, and fluctuating stresses. To create the model, the mean stress *σ*_m_ is taken into consideration. The mean stress exists when the loading is of a repeating or fluctuating type.

When considering the mean stress, the equation of the total strain is written as follows:

(6) $$\begin{array}{c} \varepsilon_{kl}=\frac{{\overset{`}{\sigma }}_{f}-\sigma_{m}}{C_{ijkl}}{\left(2N_{f}\right)}^{b}+{\overset{`}{\varepsilon }}_{f}{\left(\frac{{\overset{`}{\sigma }}_{f}-\sigma_{m}}{{\overset{`}{\sigma }}_{f}}\right)}^{c/b}{\left(2N_{f}\right)}^{c}. \end{array}$$

To find the mean stress *σ*_m_ and the ultimate stress *σ*_a_,

(7) $$\begin{array}{c} \begin{array}{c} \sigma_{m}=\frac{\sigma_{\max}+\sigma_{\min}}{2},\\ \sigma_{a}=\frac{\sigma_{\max}-\sigma_{\min}}{2}, \end{array} \end{array}$$

where *σ*_max_ and *σ*_min_ are the maximum and minimum von Mises stresses, respectively, during the loading cycle. As finding the empirical constants *b* and *c* needs experimental work, the universal slops method, shown in Figure 3(), was used instead of the Coffin-Manson relation [23]. With the universal slope method, the fatigue strength exponent (*b*) is related to the ultimate tensile strength and ductility exponent (*c*) which is related to the true strain at the fracture of the material are replaced by average slope values of −0.12 and −0.6, respectively. The total strain relation is written as follows:

(8) $$\begin{array}{c} \varepsilon_{kl}=\frac{{\overset{`}{\sigma }}_{f}-\sigma_{m}}{C_{ijkl}}{\left(2N_{f}\right)}^{-0.12}+{\overset{`}{\varepsilon }}_{f}{\left(\frac{{\overset{`}{\sigma }}_{f}-\sigma_{m}}{{\overset{`}{\sigma }}_{f}}\right)}^{-0.6/-0.12}{\left(2N_{f}\right)}^{-0.6}. \end{array}$$

And by simplifying (8),

(9) $$\begin{array}{c} \varepsilon_{kl}=\frac{{\overset{`}{\sigma }}_{f}-\sigma_{m}}{C_{ijkl}}{\left(2N_{f}\right)}^{-0.12}+{\overset{`}{\varepsilon }}_{f}{\left(\frac{{\overset{`}{\sigma }}_{f}-\sigma_{m}}{{\overset{`}{\sigma }}_{f}}\right)}^{5}{\left(2N_{f}\right)}^{-0.6}. \end{array}$$

The amount of damage experienced by the body is quantified by a single damage variable *D*. The damage variable *D* = 0 when the material is undamaged, while the damage variable *D* = 1 when the material totally failed. According to Miner's rule, the damage equation is as follows:

(10) $$\begin{array}{c} D=\overset{n}{\underset{i=0}{\sum }}\frac{n_{i}}{N_{fi}}, \end{array}$$

where *n*_*i*_ is the number of cycles of the occurred stress range and *N*_f*i*_ is the number of cycles to failure. In the case of anisotropic damage, the relation among the damage variable *D*, stress, and strain is as follows:

(11) $$\begin{array}{c} \sigma_{ij}=\left(1-D\right)C_{ijkl}\varepsilon_{kl}, \end{array}$$

where *D* denotes the damage variable, *σ*_ij_ are the stress components, *ε*_kl_ are the strains, and **C**_ijkl_ are the elasticity tensor components (stiffness matrix), where

(12) $$\begin{array}{c} \left(\begin{array}{l} \sigma_{11}\\ \sigma_{22}\\ \sigma_{33}\\ \sigma_{12}\\ \sigma_{13}\\ \sigma_{23} \end{array}\right)=\left(1-D\right)\left(\begin{array}{llllll} C_{11} & C_{12} & C_{13} & 0 & 0 & 0\\ C_{12} & C_{22} & C_{23} & 0 & 0 & 0\\ C_{13} & C_{23} & C_{33} & 0 & 0 & 0\\ 0 & 0 & 0 & C_{44} & 0 & 0\\ 0 & 0 & 0 & 0 & C_{55} & 0\\ 0 & 0 & 0 & 0 & 0 & C_{66} \end{array}\right)\left(\begin{array}{l} \varepsilon_{11}\\ \varepsilon_{22}\\ \varepsilon_{33}\\ \varepsilon_{12}\\ \varepsilon_{13}\\ \varepsilon_{23} \end{array}\right), \end{array}$$

(13) $$\begin{array}{c} \begin{array}{c} C_{11}=E_{1}\left(1-v_{23}v_{32}\right)\gamma ,\\ C_{22}=E_{2}\left(1-v_{13}v_{31}\right)\gamma ,\\ C_{33}=E_{3}\left(1-v_{12}v_{21}\right)\gamma ,\\ C_{12}=E_{1}\left(v_{21}-v_{31}v_{23}\right)\gamma =E_{2}\left(v_{12}-v_{13}v_{32}\right)\gamma ,\\ C_{13}=E_{1}\left(v_{31}-v_{21}v_{32}\right)\gamma =E_{3}\left(v_{13}-v_{23}v_{12}\right)\gamma ,\\ C_{23}=E_{2}\left(v_{32}-v_{12}v_{31}\right)\gamma =E_{3}\left(v_{23}-v_{21}v_{13}\right)\gamma ,\\ C_{44}=G_{23},\\ C_{55}=G_{31},\\ C_{66}=G_{12}, \end{array} \end{array}$$

where *E* denotes the Young's modulus, *G* denotes the shear modulus, and *ν* denotes Poisson's ratio. The superscript numbers denote the following: 1 for radial direction, 2 for circumferential direction, and 3 for longitudinal direction.

Also,

(14) $$\begin{array}{c} \Upsilon =\frac{1}{1-\upsilon_{12}\upsilon_{21}-\upsilon_{23}\upsilon_{32}-2\upsilon_{21}\upsilon_{32}\upsilon_{13}}. \end{array}$$

Table 3 shows the elasticity tensor components for each material group calculated by using (13) and (14). The proposed model of macrodamage accumulation of the bone tissue can be written as follows:

(15) $$\begin{array}{c} D=\left(\frac{\left(C_{ijkl}\varepsilon_{kl}\right)-\sigma_{ij}}{C_{ijkl}\varepsilon_{kl}}\right). \end{array}$$

And by applying (15), the final equation for damage is as follows:

(16) $$\begin{array}{c} D=\left(\frac{\left(C_{ijkl}\left(\left(\left({\overset{`}{\sigma }}_{f}-\sigma_{m}\right)/\left(C_{ijkl}\right)\right){\left(2N_{f}\right)}^{-0.12}+{\overset{`}{\varepsilon }}_{f}{\left(\left({\overset{`}{\sigma }}_{f}-\sigma_{m}\right)/\left({\overset{`}{\sigma }}_{f}\right)\right)}^{5}{\left(2N_{f}\right)}^{-0.6}\right)\right)-\sigma_{ij}}{C_{ijkl}\left(\left(\left({\overset{`}{\sigma }}_{f}-\sigma_{m}\right)/\left(C_{ijkl}\right)\right){\left(2N_{f}\right)}^{-0.12}+{\overset{`}{\varepsilon }}_{f}{\left(\left({\overset{`}{\sigma }}_{f}-\sigma_{m}\right)/\left({\overset{`}{\sigma }}_{f}\right)\right)}^{5}{\left(2N_{f}\right)}^{-0.6}\right)}\right). \end{array}$$

A single scalar damage variable is often insufficient to describe the variation in mechanical properties of damaged materials.

#### 2.2.1. Applicability of Damage Models to the Femur

**Figure 14:**
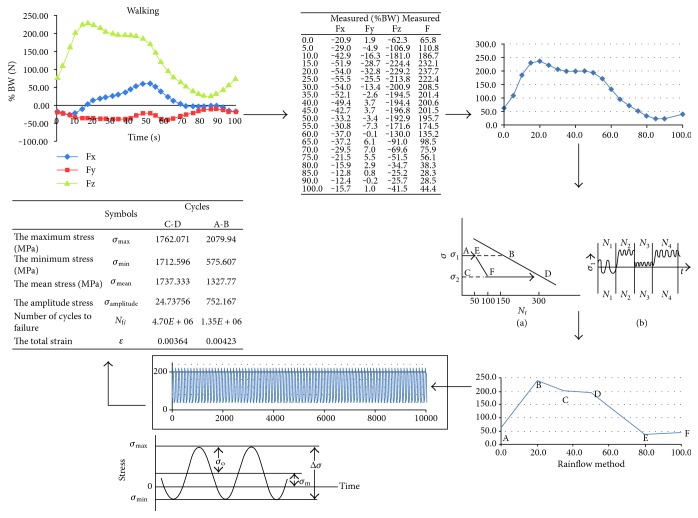
The procedure of applying the rainflow method. Firstly, the Fx, Fy, and Fz forces have been simplified into one curve. Then, the rainflow method [22] has been applied to simplify the counting of the loading cycles. Finally, the measurements have been done for 10^6^ cycles.

The gait cycle of the hip is used to predict the macrodamage accumulation for the femoral bone. Because the femoral bone is subjected to a complex loading, the rainflow method is used to simplify the counting of load cycles. This method is very accommodating with the use of Miner's rule. The values of strength and ductility coefficients were used from the literature. The value of fatigue strength coefficient ${\overset{`}{\sigma }}_{f}$ that was used is 6, and the fatigue ductility coefficient ${\overset{`}{\varepsilon }}_{f}$ value that was used is 0.352 [24]. The procedure of using the rainflow method is shown in Figure 14.

A comparison between the proposed model and the three different macrodamage accumulations models was performed. The first model was for the cortical bone only, the second model for the trabecular bone only, and the third model for both cortical and trabecular composite bones. The first model is for the damage of the cortical bone from Pattin et al. [25]. In their study, thirty-two specimens of the cortical bone were used; the stress range (∆*σ*) = 83 MPa, number of cycles to failure (*N*_f_) = 417, and the modulus (*E*_f_) = 9.02 GPa. The other model is for the trabecular bone from Hambli [13]. In his study, five specimens were taken from the trabecular part of the head of the femoral bone; the stress range (∆*σ*) = 85 MPa, number of cycles to failure (*N*_f_) = 10^7^, and the modulus (*E*_f_) = 0.17 GPa. The third model is for the damage of both the cortical and trabecular bone components from Zioupos and Casinos [26]. On the other hand, Miner's rule and the finite element analysis data were used for the proposed model of the femoral bone that has both the cortical and trabecular components.

**Figure 15:**
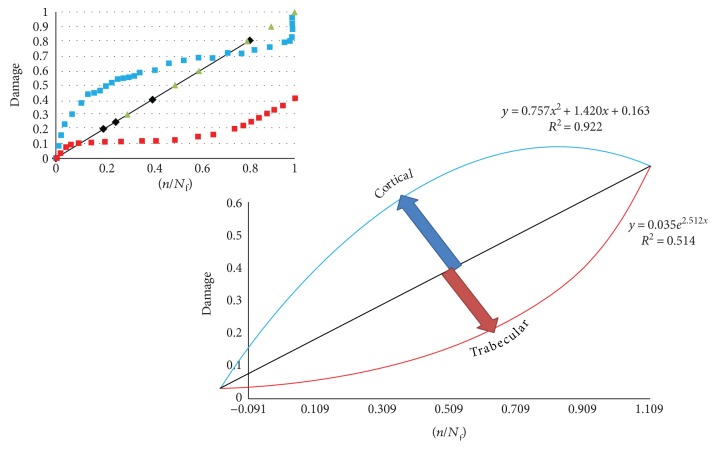
The relation between the damage of the bone and the fraction of fatigue lifetime cycles (*n/N*_f_) (cortical bone, Pattin et al. [25], and trabecular bone, Hambli [13]).

**Figure 16:**
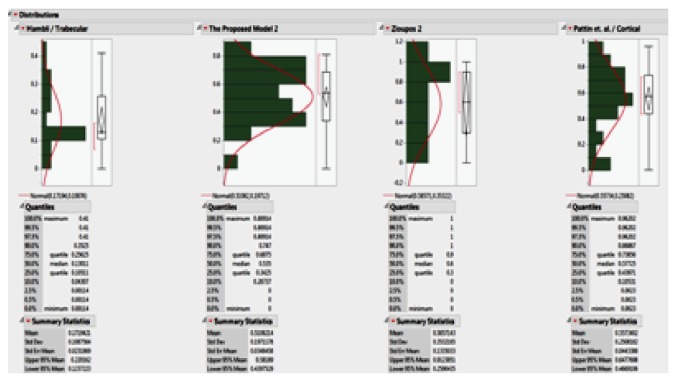
Monte Carlo simulation of the four models using the JMP program.

Figure 15 shows the relation between the damages of the bones in terms of cycle fraction (*n/N*_f_) for the models, where *n* is the number of cycles at a specific stress range and *N*_f_ is the number of cycles to failure at the same stress range. The convex curve shows the damage of the cortical bone, while the concave curve shows the damage accumulation of the trabecular bone as the cycles increase. Monte Carlo simulation was performed using the results from deterministic analysis that shows damage accumulation with number of cycles, probabilistically. Monte Carlo simulation generates a set of random variables normally distributed about a mean and standard deviation. Monte Carlo simulation was carried out for the proposed model and the other three models. The mean and standard deviation for each macrodamage accumulation model have been measured by using the JMP program, as shown in Figure 16. The simulation for each model consisted of 200 random generated variables normally distributed. The probability of failure was calculated for each model.  Table 4 shows the mean, standard deviation (SD), variance, and probability of failure for the four models.

## 3. Results

**Figure 17:**
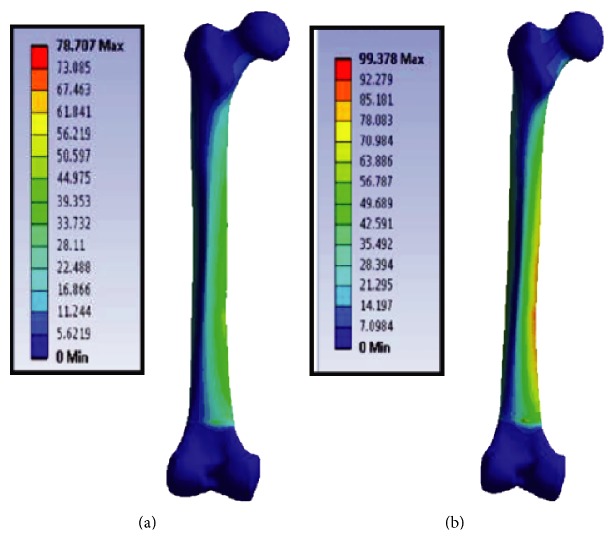
von Mises stresses for the static uniaxial (a) and multiaxial (b) loading conditions.

**Figure 18:**
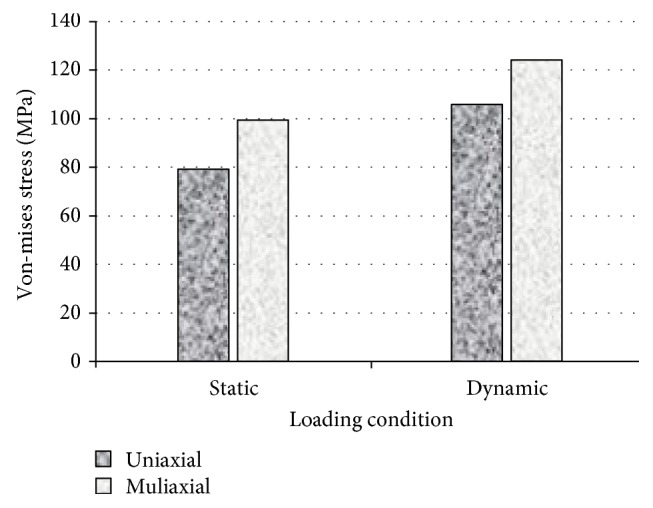
Finite element simulation results for both the static and dynamic loading conditions.

**Figure 19:**
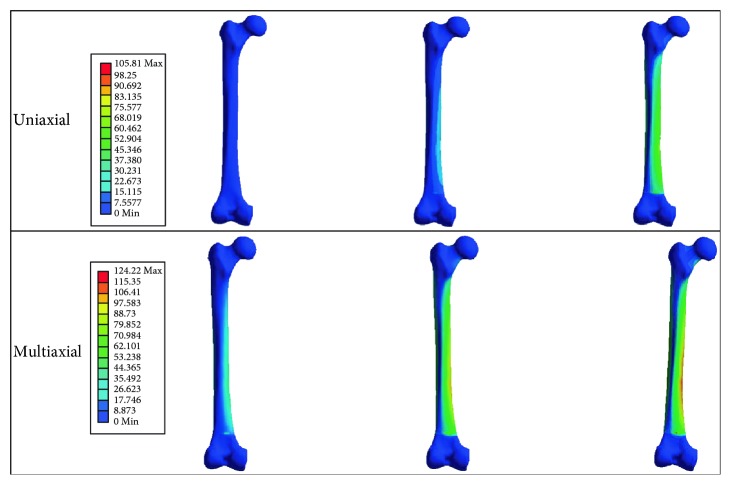
von Mises stresses for the dynamic uniaxial and multiaxial loading conditions for different cycles.

**Figure 20:**
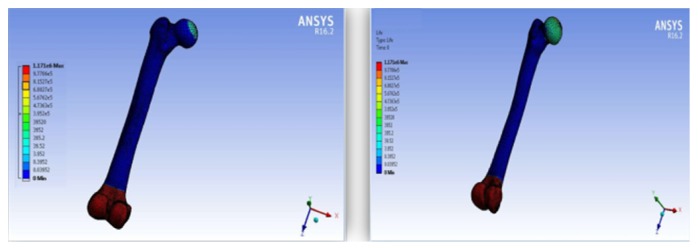
Total life for the uniaxial and multiaxial loading conditions; the results show that the bone is not a synthetic material with regeneration/remodeling capabilities.

**Figure 21:**
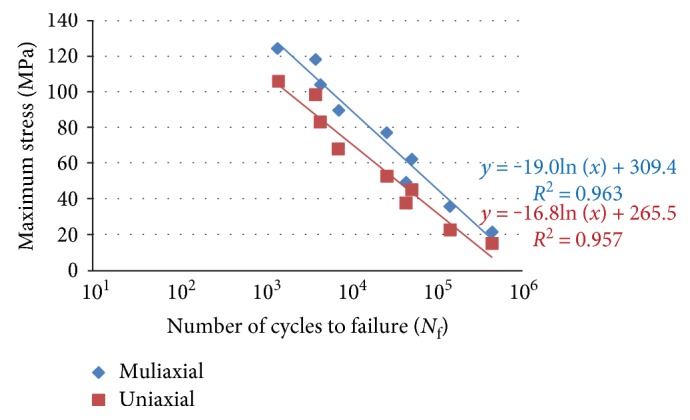
Maximum stress versus the number of cycles to failure for both the uniaxial and the multiaxial dynamic loading conditions.

**Figure 22:**
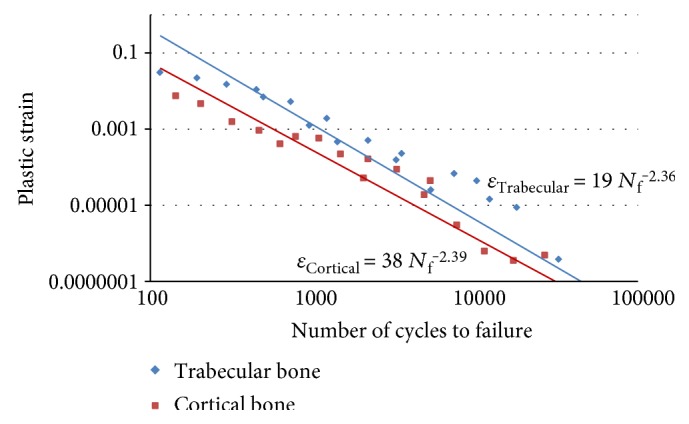
Plastic strain versus the number of cycles to failure for the cortical bone and the trabecular bone. Equation (6) was used to calculate the strains. The measurements of the stresses and the number of cycles were obtained from the finite element simulation.

First, stress-strain analyses in uniaxial and multiaxial loading conditions are considered, then fatigue life prediction of the bone is carried out. The maximum von Mises stresses were obtained from both uniaxial and multiaxial loading conditions for static simulations, as shown in Figures 17 and 18, where the stresses are 78.7 and 99.4 MPa for the uniaxial and multiaxial loadings, respectively.  Figure 19 shows the von Mises stresses for the dynamic simulation of both loading conditions, where the stresses are 105.8 and 124.2 MPa for the uniaxial and multiaxial loadings, respectively. In addition, the total life was obtained from both uniaxial and multiaxial loading conditions for the dynamic simulation assuming that the bone is not a synthetic material with regeneration/remodeling capabilities, as shown in Figure 20. The relation between the maximum stress and the number of cycles to failure is shown in Figure 21 for both the uniaxial and multiaxial loading conditions. The polynomial curve fitting (*σ*_max_ = −19.0  ln  (*N*_f_) + 309.4, *R*^2^ = 0.963 for the multiaxial loading condition, and *σ*_max_ = −16.8  ln  (*N*_f_) + 265.5, *R*^2^ = 0.957 for the uniaxial loading condition) proves that the stress decreases linearly with the increase in life or number of cycles to failure (*N*_f_). In addition, Figure 22 shows that for the given life, the trabecular bone accumulated approximately 25% more plastic strain than the cortical bone. Also, the same trend was observed with elastic strain accumulation in the trabecular bone where it was approximately 6% higher than the cortical bone.

**Figure 23:**
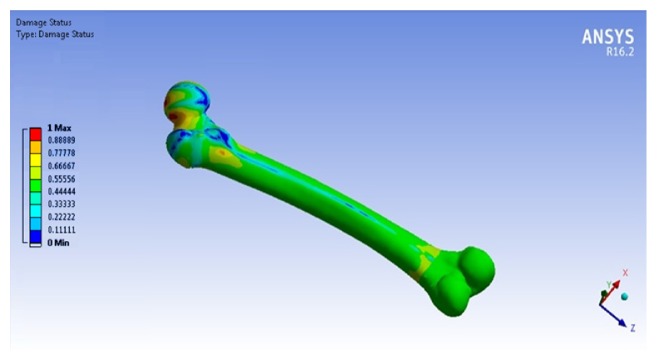
The macrodamage of the femoral bone under 10^6^ cycles.

**Figure 24:**
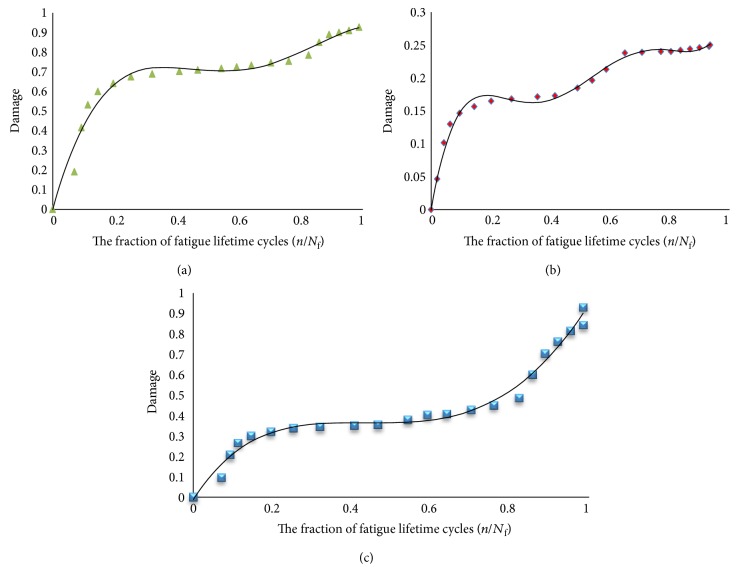
The relation between the fraction of fatigue lifetime (*n*/*N*_f_) and the damage of (a) the cortical bone, (b) the trabecular bone, and (c) the combined model that has both.

The finite element modeling of damage considers that the damage equals to zero when the element in the region of interest is undamaged. While, the damage is equal to one when the element failed.  Figure 23 shows that the damage starts at the femoral neck after 10^6^ cycles. To make a comparison between the cortical and trabecular components of the bone, each part has been evaluated individually.  Figure 24 shows the relation between the damage and the fraction of fatigue lifetime (*n*/*N*_f_).

Force versus displacement curves were presented from the finite element analysis, as shown in Figure 5(). The polynomial curves fitting for the whole bone data (*F* = 562.9d^3^–1461d^2^ + 6538d, *R*^2^ = 0.996), for the cortical bone (*F* = −1284d^3^–8091d^2^ + 20430d, *R*^2^ = 0.994), and for the trabecular bone (*F* = 1029d^3^–798.1d^2^ + 2170d, *R*^2^ = 0.997) suggest linear relation between the force and the displacement.

To measure the stiffness, data was generated from 26,898 elements and analyzed. It appears that the mean stiffness of the cortical bone was 7890; trabecular bone, 2860; and that of the whole bone, 4864 N/mm, as expected.

## 4. Discussion

Macrodamage accumulation of bone tissue was estimated for two different loading conditions. For the mathematical part, the elastic and plastic behaviors of the bone were taken into consideration. Also, the anisotropic and the nonhomogeneous material properties of the cortical and trabecular zones were included. The MIMICS program was used to create the material properties depending on the Hounsfield unit and the relation among the density of the bone and the modulus of elasticity and Poisson's ratio assigned based on grayscale distribution across the 3D model of the femur.

To validate the importance of studying the bone as a composite material, a study on each part of the bone has been done separately also. This is very important in order to understand the effect of each zone on the whole bone. A comparison between the proposed model and the three different macrodamage accumulations models was performed. The first model [25] shows cortical bone behavior, and the study was done on a small sample of the femoral bone. The second model [13] was for the trabecular bone only, on a small sample of the bone. The third model [26] was for a portion of the bone that contained both cortical and trabecular parts. However, the material properties were simple, isotropic, and homogeneous for all the three models. Moreover, the macrodamage models were nonlinear in the first two models and linear in the last model. In the current study, Miner's rule was used with the proposed femoral model that contained both the cortical and the trabecular components, and a linear relationship was assumed. Also, the rainflow method was used to simplify the gait cycle of normal walking. Figure 15 shows that the damage in the cortical bone is higher than that in the trabecular bone for the same fraction of fatigue cycles (*n/N*_f_) at a particular stress range. For the cortical bone, the damage starts to decrease when (*n/N*_f_) reaches 0.9, while for the trabecular bone, the damage keeps increasing till (*n/N*_f_) reaches 1.

The probability of failure was calculated from the distribution of the random variables for each model by using Monte Carlo simulation. The probability of failure for the proposed model was 13.26%, while the probability of failure was 37.90% for the whole bone model and 42.20% for the cortical bone model. The reason for this large difference between the probability of failure of the proposed model and the other models is likely due to the entire femoral bone was studied in our study. On the other hand, the other models were on a small sample of the bone. The data clearly shows that the composite bone as considered in the present study has lower von Mises stresses and thus lower failure probability than elastic/plastic materials.

Furthermore, the finite element analysis allowed a deeper understanding for the macrodamage accumulation of bone tissue. A comparison between different loading conditions was evaluated. The first loading condition was a multiaxial loading, where the cycle for normal walking was used including Fx, Fy, and Fz; the other loading condition was the uniaxial loading, where the equivalent maximum stress from the gait cycle was converted into a single load cycle. The results showed a significant difference between the two loading conditions. In static finite element simulation, the maximum von Mises stress was 78 MPa for the uniaxial loading condition and 99 MPa for the multiaxial loading condition, respectively. These results were expected as the loads are higher in the multiaxial loading condition, which led to a greater amount of stress than those in the uniaxial loading condition. The advantage of the static simulation in this study is to confirm the validation of the 3D model of the femoral bone with the literature. In the dynamic finite element simulation, the maximum von Mises stresses were 105.8 MPa for the uniaxial loading condition and 124.2 MPa for the multiaxial loading condition, respectively.

The study showed that the failure starts faster in the multiaxial loading condition than that in the uniaxial loading condition for the same number of cycles. Furthermore, the finite element simulation showed that the relation between the stress and the strain stays the same till the stress reaches 65 MPa. Then, the stress starts to be higher for the multiaxial loading condition than that for the uniaxial loading condition for the same amount of displacement. In addition, the finite element simulation for the damage of the bone showed that the damage starts at the femoral neck. This result was expected, as the femoral neck is the weakest point in the femoral bone, and the study was done on a healthy bone that does not have any injury.

The anisotropic material properties were used in the finite element simulation of the proposed model. The damage accumulation process in a long bone may be described by a three-stage process, as shown in Figure 26.

Since stage II shows a linear behavior, stage I is reflective of the primary phase, where the damage developed in the cortical bone decreases as the cycle fractions increase. However, as stage I transitions to stage II, the damage accumulated in the cortical bone increases linearly until about a cycle fraction of 0.8; upon attaining this level of fatigue life, the damage mode transitions to a more rapid damage accumulation that cannot be described by a linear equation. This state, stage III, is known as the tertiary damage accumulation stage and must lead to the bone fracture. We proposed this behavior for the cortical bone, and the lower ranges of damage hold good for the trabecular bone as well, assuming that the bone is anisotropic and nonhomogeneous. However, the damage accumulated on the composite bone was derived from the material properties of both the cortical and trabecular bones. Three damage prediction equations were developed, as shown in Table 5, where *B* represents the fatigue cycle fractions (*n/N*_f_) at a particular stress range. These equations can be used in deriving the bone fracture at a given stress range and fatigue life. The charts in Figure 26 show that *R*^2^ decreases as the damage increases. The failure starts in the cortical bone before the trabecular bone.

By comparing between the behaviors of the damage of the bone that were reported in Figure 15 versus Figure 27, our effort shows a very clear three-stage process. Therefore, the mathematical significance of our analysis is applicable in the engineering design.

**Figure 25:**
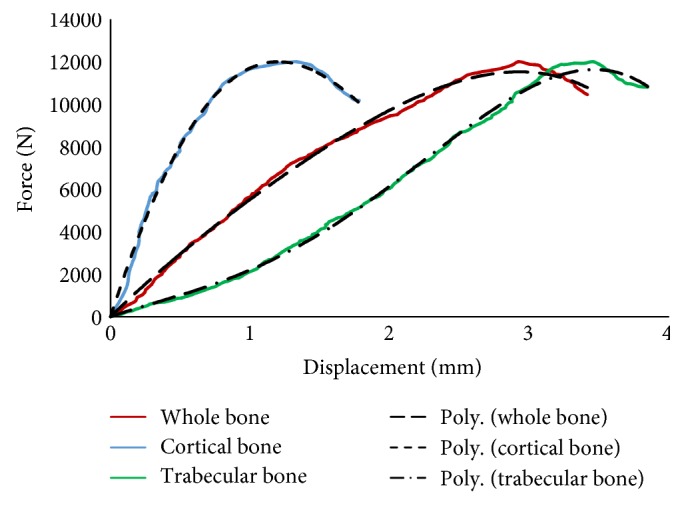
Force (N) versus displacement (mm) curves (the data from the finite element simulation).

**Figure 26:**
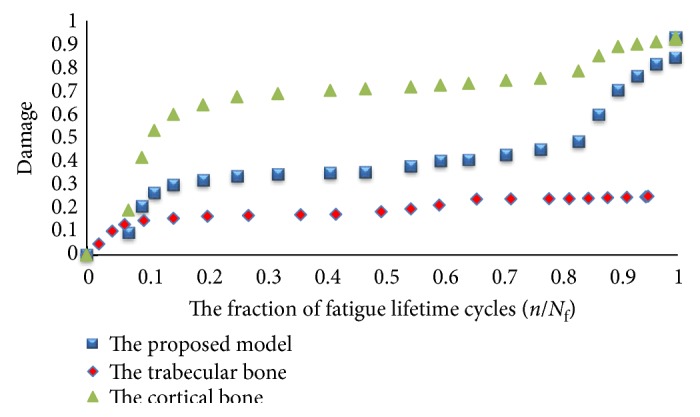
The relation between the damage of the cortical, trabecular, and both of them and the fraction of fatigue lifetime (*n/N*_f_); the finite element results were used here to check the validity of the proposed model.

**Figure 27:**
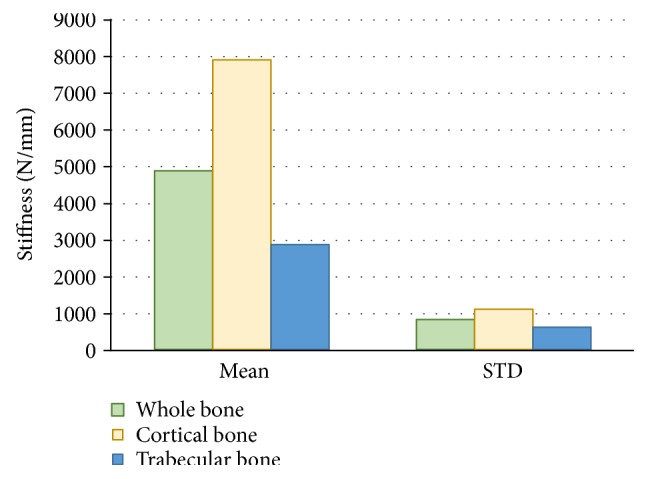
Whole bone, cortical bone, and trabecular bone stiffness (N/mm) results.

The results from the FE analysis was used to determine mean stiffness. It appears that the mean stiffness of the cortical bone was 7890; trabecular bone, 2860; and that of whole bone, 4864 N/mm. Data generated from 26,898 elements was analyzed, and we observed a significant difference in the stiffness of each element. The stiffness is observed in Figures 25 and 27 for the whole bone and the cortical and trabecular bone components, respectively. The micromotions or displacements in the hip with implants were investigated [27, 28] and found to be 2.5 to 6 times higher in the composite bone than with the implants. This difference was a result of the mismatch between the *E* values of the bone and implant materials.

Our results are consistent with stress concentration on the bone surface via the body and surface stress. These stresses are concentrated on the first layers of the cortical bone which is several millimeters thick. Since we are assuming repeated cyclic loads in this study, damage likely concentrated on the surface comprised of the cortical bone. Since mechanism in the cancellous bone is displacement driven, the composite bone assumes that stress on both the zones will be same whereas the displacement will be different. Also, our results are consistent with femoral fractures observed clinically resulting from high stress.

The results of FEM analysis is presented in terms of both max von Mises stress and strain values (Figures 9() and 0()), respectively, showing the composite laws and material properties as expected, that is, the displacement in the trabecular zone is higher, resulting in a higher strain than that in the cortical zone. Also, a higher total strain obtained life for the *D* equivalent of 1 is lower than at low total strains. A similar trend was found for the von Mises stress plot as well.

The limitations of this study can be the inability to validate the in vivo conditions in the absence of a biological self-healing environment. The second limitation is the computer, which makes it harder to apply more than 10^6^ cycles during the damage simulation of the bone.

## 5. Conclusion

1. Based on the nonlinear relationship of the macrodamage mathematical models of bone tissue, a conceptual model has been proposed and tested on a human femur. Monte Carlo simulation showed that the probability of failure for the proposed model was lower than that for the other models. The reason for this difference is that in this study the entire femoral bone was separated in terms of cortical and trabecular components.
2. The results have been validated using anisotropic material properties that showed the bone tissue damage cannot be expressed by only the cortical or the trabecular bone and both of them should be taken into consideration to develop a more realistic simulation.
3. Three damage prediction equations were developed (cortical, trabecular, and together cortical and trabecular). These equations can be used in deriving the bone fracture equations at a given stress range and fatigue life.
4. The study showed that the failure starts faster in the multiaxial loading condition than the uniaxial loading condition for the same number of cycles in the finite element simulation. Also, the damage starts at the femoral neck, as the femoral neck is the weakest part of the femoral bone.
5. The failure starts in the cortical bone before the trabecular bone. This means that the trabecular bone is more ductile while the cortical bone is more brittle.
6. The damage behavior seems to follow a three-stage regression; stage one was described by the primary phase of damage growth, stage two was described by the secondary phase of damage growth, and stage three was described by the tertiary phase of damage growth.
7. There is a significant difference in the stiffness of each element. Also, the stiffness of the cortical bone and the trabecular bone are significantly different as expected.

## Figures and Tables

**Figure 13:**
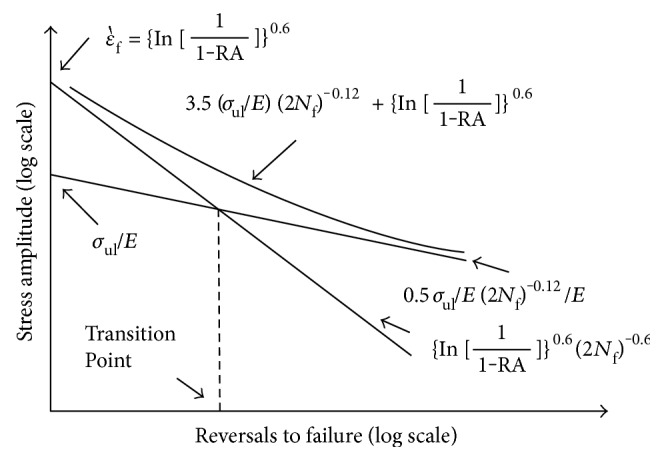
Universal slope method stress-life curve [23].

**Table 1:** The orthotropic relationships between elastic constants and density.

	Modulus of elasticity	Poisson ratio	Shear modulus
Cortical bone	*E*_1_ = 2314*ρ*^1.57^	*ν*_12_ = 0.4	*G*_12_ = (*G*_12 max_*ρ*^2^)/(*ρ*_max_^2^)
	*E*_2_ = 2314*ρ*^1.57^	*ν*_23_ = 0.25	*G*_23_ = (*G*_23 max_*ρ*^2^)/(*ρ*_max_^2^)
	*E*_3_ = 2065*ρ*^3.09^	*ν*_31_ = 0.25	*G*_31_ = (*G*_31 max_*ρ*^2^)/(*ρ*_max_^2^)
Trabecular bone	*E*_1_ = 1157*ρ*^1.78^	*ν*_12_ = 0.4	*G*_12_ = (*G*_12 max_*ρ*^2^)/(*ρ*_max_^2^)
	*E*_2_ = 1157*ρ*^1.78^	*ν*_23_ = 0.25	*G*_23_ = (*G*_23 max_*ρ*^2^)/(*ρ*_max_^2^)
	*E*_3_ = 1904*ρ*^1.64^	*ν*_31_ = 0.25	*G*_31_ = (*G*_31 max_*ρ*^2^)/(*ρ*_max_^2^)

*ρ*_max_ represents the maximum density, *G*_12 max_ = 5.71 MPa, *G*_23 max_ = 7.11 MPa, and *G*_31 max_ = 6.58 MPa. The superscript numbers denote the following: 1 for the radial direction, 2 for the circumferential direction, and 3 for the longitudinal direction [14–16].

**Table 2:** Material group numbers with their densities and the nine elastic constants. The subscript numbers denote the following: 1 for the radial direction, 2 for the circumferential direction, and 3 for the longitudinal direction.

Material group number	Density (g/cm^3^)	*E*_1_ (GPa)	*E*_2_ (GPa)	*E*_3_ (GPa)	*ν*_12_	*ν*_23_	*ν*_31_	*G*_12_	*G*_23_	*G*_31_
1	0.997	1.151	1.151	1.894	0.40	0.25	0.25	0.053	0.066	0.061
2	0.998	1.153	1.153	1.897	0.40	0.25	0.25	0.053	0.066	0.061
3	0.999	1.154	1.154	1.900	0.40	0.25	0.25	0.053	0.066	0.061
4	1.000	1.156	1.156	1.903	0.40	0.25	0.25	0.053	0.066	0.061
5	1.001	1.158	1.158	1.906	0.40	0.25	0.25	0.053	0.066	0.061
6	1.002	1.160	1.160	1.909	0.40	0.25	0.25	0.053	0.066	0.061
7	1.002	1.162	1.162	1.912	0.40	0.25	0.25	0.053	0.066	0.061
8	1.003	1.164	1.164	1.915	0.40	0.25	0.25	0.053	0.067	0.062
9	1.004	1.166	1.166	1.918	0.40	0.25	0.25	0.054	0.067	0.062
10	1.005	1.168	1.168	1.921	0.40	0.25	0.25	0.054	0.067	0.062
11	1.006	1.170	1.170	1.923	0.40	0.25	0.25	0.054	0.067	0.062
12	1.007	1.172	1.172	1.926	0.40	0.25	0.25	0.054	0.067	0.062
13	1.008	1.174	1.174	1.929	0.40	0.25	0.25	0.054	0.067	0.062
14	1.009	1.176	1.176	1.932	0.40	0.25	0.25	0.054	0.067	0.062
15	1.010	1.178	1.178	1.935	0.40	0.25	0.25	0.054	0.067	0.062
16	1.011	1.180	1.180	1.938	0.40	0.25	0.25	0.054	0.068	0.063
17	1.012	1.181	1.181	1.941	0.40	0.25	0.25	0.054	0.068	0.063
18	1.013	1.183	1.183	1.944	0.40	0.25	0.25	0.054	0.068	0.063
19	1.014	1.185	1.185	1.947	0.40	0.25	0.25	0.055	0.068	0.063
20	1.015	1.187	1.187	1.950	0.40	0.25	0.25	0.055	0.068	0.063
21	1.016	1.189	1.189	1.953	0.40	0.25	0.25	0.055	0.068	0.063
22	1.016	1.191	1.191	1.956	0.40	0.25	0.25	0.055	0.068	0.063
23	1.017	1.193	1.193	1.959	0.40	0.25	0.25	0.055	0.068	0.063
24	1.018	1.195	1.195	1.962	0.40	0.25	0.25	0.055	0.069	0.063
25	1.019	1.197	1.197	1.965	0.40	0.25	0.25	0.055	0.069	0.064
26	1.020	1.199	1.199	1.968	0.40	0.25	0.25	0.055	0.069	0.064
27	1.021	1.201	1.201	1.971	0.40	0.25	0.25	0.055	0.069	0.064
28	1.022	1.203	1.203	1.973	0.40	0.25	0.25	0.055	0.069	0.064
29	1.023	1.205	1.205	1.976	0.40	0.25	0.25	0.056	0.069	0.064
30	1.024	1.207	1.207	1.979	0.40	0.25	0.25	0.056	0.069	0.064
31	1.025	1.209	1.209	1.982	0.40	0.25	0.25	0.056	0.069	0.064
32	1.026	1.211	1.211	1.985	0.40	0.25	0.25	0.056	0.070	0.064
33	1.027	1.213	1.213	1.988	0.40	0.25	0.25	0.056	0.070	0.065
34	1.028	1.215	1.215	1.991	0.40	0.25	0.25	0.056	0.070	0.065
35	1.029	1.217	1.217	1.994	0.40	0.25	0.25	0.056	0.070	0.065
36	1.030	1.219	1.219	1.997	0.40	0.25	0.25	0.056	0.070	0.065
37	1.030	1.221	1.221	2.000	0.40	0.25	0.25	0.056	0.070	0.065
38	1.031	1.223	1.223	2.003	0.40	0.25	0.25	0.056	0.070	0.065
39	1.032	1.224	1.224	2.006	0.40	0.25	0.25	0.057	0.070	0.065
40	1.033	1.226	1.226	2.009	0.40	0.25	0.25	0.057	0.071	0.065
41	1.034	1.228	1.228	2.012	0.40	0.25	0.25	0.057	0.071	0.065
42	1.035	1.230	1.230	2.015	0.40	0.25	0.25	0.057	0.071	0.066
43	1.036	1.232	1.232	2.018	0.40	0.25	0.25	0.057	0.071	0.066
44	1.037	1.234	1.234	2.021	0.40	0.25	0.25	0.057	0.071	0.066
45	1.038	12.363	12.363	20.240	0.40	0.25	0.25	5.367	6.683	6.185
46	1.039	12.383	12.383	20.269	0.40	0.25	0.25	5.377	6.695	6.196
47	1.040	12.403	12.403	20.299	0.40	0.25	0.25	5.386	6.707	6.207
48	1.041	12.423	12.423	20.329	0.40	0.25	0.25	5.396	6.719	6.218
49	1.042	12.442	12.442	20.359	0.40	0.25	0.25	5.406	6.731	6.229
50	1.043	12.462	12.462	20.389	0.40	0.25	0.25	5.415	6.743	6.240
51	1.044	12.482	12.482	20.419	0.40	0.25	0.25	5.425	6.755	6.252
52	1.044	12.502	12.502	20.449	0.40	0.25	0.25	5.435	6.767	6.263
53	1.045	12.522	12.522	20.479	0.40	0.25	0.25	5.444	6.779	6.274
54	1.046	12.542	12.542	20.509	0.40	0.25	0.25	5.454	6.791	6.285
55	1.047	12.562	12.562	20.539	0.40	0.25	0.25	5.464	6.804	6.296
56	1.048	12.582	12.582	20.569	0.40	0.25	0.25	5.474	6.816	6.308
57	1.049	12.602	12.602	20.599	0.40	0.25	0.25	5.483	6.828	6.319
58	1.050	12.622	12.622	20.629	0.40	0.25	0.25	5.493	6.840	6.330
59	1.051	12.642	12.642	20.659	0.40	0.25	0.25	5.503	6.852	6.341
60	1.052	12.662	12.662	20.689	0.40	0.25	0.25	5.513	6.864	6.353
61	1.053	12.682	12.682	20.719	0.40	0.25	0.25	5.522	6.876	6.364
62	1.054	12.702	12.702	20.749	0.40	0.25	0.25	5.532	6.889	6.375
63	1.055	12.722	12.722	20.779	0.40	0.25	0.25	5.542	6.901	6.386
64	1.056	12.742	12.742	20.810	0.40	0.25	0.25	5.552	6.913	6.398
65	1.057	12.762	12.762	20.840	0.40	0.25	0.25	5.562	6.925	6.409
66	1.058	12.782	12.782	20.870	0.40	0.25	0.25	5.572	6.938	6.420
67	1.058	12.802	12.802	20.900	0.40	0.25	0.25	5.581	6.950	6.432
68	1.059	12.822	12.822	20.930	0.40	0.25	0.25	5.591	6.962	6.443
69	1.060	12.842	12.842	20.961	0.40	0.25	0.25	5.601	6.974	6.454
70	1.061	12.862	12.862	20.991	0.40	0.25	0.25	5.611	6.987	6.466
71	1.062	12.882	12.882	21.021	0.40	0.25	0.25	5.621	6.999	6.477
72	1.063	12.902	12.902	21.051	0.40	0.25	0.25	5.631	7.011	6.489
73	1.064	12.923	12.923	21.082	0.40	0.25	0.25	5.641	7.024	6.500
74	1.065	12.943	12.943	21.112	0.40	0.25	0.25	5.650	7.036	6.511
75	1.066	12.963	12.963	21.142	0.40	0.25	0.25	5.660	7.048	6.523
76	1.067	12.983	12.983	21.173	0.40	0.25	0.25	5.670	7.061	6.534
77	1.068	13.003	13.003	21.203	0.40	0.25	0.25	5.680	7.073	6.546
78	1.069	13.024	13.024	21.234	0.40	0.25	0.25	5.690	7.085	6.557
79	1.070	13.044	13.044	21.264	0.40	0.25	0.25	5.700	7.098	6.569
80	1.071	13.064	13.064	21.294	0.40	0.25	0.25	5.710	7.110	6.580

**Table 3:** Material group numbers with the elasticity tensor components (stiffness matrix). The subscript numbers denote the following: 1 for the radial direction, 2 for the circumferential direction, and 3 for the longitudinal direction.

Material group number	Density (g/cm^3^)	**C**_11_	**C**_22_	**C**_33_	**C**_12_	**C**_13_	**C**_23_	**C**_44_	**C**_55_	**C**_66_
1	0.997	1.423	1.483	2.187	0.534	0.297	0.237	0.066	0.061	0.053
2	0.998	1.426	1.541	2.455	0.535	0.297	0.238	0.066	0.061	0.053
3	0.999	1.428	1.546	2.467	0.536	0.298	0.238	0.066	0.061	0.053
4	1.000	1.431	1.551	2.479	0.536	0.298	0.238	0.066	0.061	0.053
5	1.001	1.433	1.556	2.491	0.537	0.299	0.239	0.066	0.061	0.053
6	1.002	1.435	1.561	2.503	0.538	0.299	0.239	0.066	0.061	0.053
7	1.002	1.438	1.566	2.516	0.539	0.300	0.240	0.066	0.061	0.053
8	1.003	1.440	1.572	2.528	0.540	0.300	0.240	0.067	0.062	0.053
9	1.004	1.442	1.577	2.540	0.541	0.301	0.240	0.067	0.062	0.054
10	1.005	1.445	1.582	2.552	0.542	0.301	0.241	0.067	0.062	0.054
11	1.006	1.447	1.587	2.565	0.543	0.302	0.241	0.067	0.062	0.054
12	1.007	1.450	1.593	2.577	0.544	0.302	0.242	0.067	0.062	0.054
13	1.008	1.452	1.598	2.589	0.545	0.303	0.242	0.067	0.062	0.054
14	1.009	1.454	1.603	2.602	0.545	0.303	0.242	0.067	0.062	0.054
15	1.010	1.457	1.608	2.614	0.546	0.304	0.243	0.067	0.062	0.054
16	1.011	1.459	1.614	2.627	0.547	0.304	0.243	0.068	0.063	0.054
17	1.012	1.462	1.619	2.640	0.548	0.305	0.244	0.068	0.063	0.054
18	1.013	1.464	1.624	2.652	0.549	0.305	0.244	0.068	0.063	0.054
19	1.014	1.466	1.630	2.665	0.550	0.306	0.244	0.068	0.063	0.055
20	1.015	1.469	1.635	2.678	0.551	0.306	0.245	0.068	0.063	0.055
21	1.016	1.471	1.640	2.691	0.552	0.307	0.245	0.068	0.063	0.055
22	1.016	1.474	1.646	2.704	0.553	0.307	0.246	0.068	0.063	0.055
23	1.017	1.476	1.651	2.717	0.554	0.308	0.246	0.068	0.063	0.055
24	1.018	1.478	1.656	2.729	0.554	0.308	0.246	0.069	0.063	0.055
25	1.019	1.481	1.662	2.743	0.555	0.309	0.247	0.069	0.064	0.055
26	1.020	1.483	1.667	2.756	0.556	0.309	0.247	0.069	0.064	0.055
27	1.021	1.486	1.673	2.769	0.557	0.310	0.248	0.069	0.064	0.055
28	1.022	1.488	1.678	2.782	0.558	0.310	0.248	0.069	0.064	0.055
29	1.023	1.491	1.684	2.795	0.559	0.311	0.248	0.069	0.064	0.056
30	1.024	1.493	1.689	2.808	0.560	0.311	0.249	0.069	0.064	0.056
31	1.025	1.495	1.695	2.822	0.561	0.312	0.249	0.069	0.064	0.056
32	1.026	1.498	1.700	2.835	0.562	0.312	0.250	0.070	0.064	0.056
33	1.027	1.500	1.706	2.849	0.563	0.313	0.250	0.070	0.065	0.056
34	1.028	1.503	1.711	2.862	0.563	0.313	0.250	0.070	0.065	0.056
35	1.029	1.505	1.717	2.876	0.564	0.314	0.251	0.070	0.065	0.056
36	1.030	1.508	1.722	2.889	0.565	0.314	0.251	0.070	0.065	0.056
37	1.030	1.510	1.728	2.903	0.566	0.315	0.252	0.070	0.065	0.056
38	1.031	1.512	1.733	2.917	0.567	0.315	0.252	0.070	0.065	0.056
39	1.032	1.515	1.739	2.930	0.568	0.316	0.252	0.070	0.065	0.057
40	1.033	1.517	1.745	2.944	0.569	0.316	0.253	0.071	0.065	0.057
41	1.034	1.520	1.750	2.958	0.570	0.317	0.253	0.071	0.065	0.057
42	1.035	1.522	1.756	2.972	0.571	0.317	0.254	0.071	0.066	0.057
43	1.036	1.525	1.761	2.986	0.572	0.318	0.254	0.071	0.066	0.057
44	1.037	1.527	1.767	3.000	0.573	0.318	0.255	0.071	0.066	0.057
45	1.038	15.295	15.932	23.369	5.736	3.186	2.549	6.683	6.185	5.367
46	1.039	15.319	15.958	23.404	5.745	3.192	2.553	6.695	6.196	5.377
47	1.040	15.344	15.983	23.438	5.754	3.197	2.557	6.707	6.207	5.386
48	1.041	15.368	16.009	23.473	5.763	3.202	2.561	6.719	6.218	5.396
49	1.042	15.393	16.034	23.507	5.772	3.207	2.565	6.731	6.229	5.406
50	1.043	15.417	16.060	23.542	5.781	3.212	2.570	6.743	6.240	5.415
51	1.044	15.442	16.085	23.576	5.791	3.217	2.574	6.755	6.252	5.425
52	1.044	15.466	16.111	23.611	5.800	3.222	2.578	6.767	6.263	5.435
53	1.045	15.491	16.137	23.646	5.809	3.227	2.582	6.779	6.274	5.444
54	1.046	15.516	16.162	23.680	5.818	3.232	2.586	6.791	6.285	5.454
55	1.047	15.540	16.188	23.715	5.828	3.238	2.590	6.804	6.296	5.464
56	1.048	15.565	16.214	23.750	5.837	3.243	2.594	6.816	6.308	5.474
57	1.049	15.590	16.239	23.784	5.846	3.248	2.598	6.828	6.319	5.483
58	1.050	15.614	16.265	23.819	5.855	3.253	2.602	6.840	6.330	5.493
59	1.051	15.639	16.291	23.854	5.865	3.258	2.607	6.852	6.341	5.503
60	1.052	15.664	16.316	23.888	5.874	3.263	2.611	6.864	6.353	5.513
61	1.053	15.689	16.342	23.923	5.883	3.268	2.615	6.876	6.364	5.522
62	1.054	15.713	16.368	23.958	5.892	3.274	2.619	6.889	6.375	5.532
63	1.055	15.738	16.394	23.993	5.902	3.279	2.623	6.901	6.386	5.542
64	1.056	15.763	16.420	24.028	5.911	3.284	2.627	6.913	6.398	5.552
65	1.057	15.788	16.445	24.062	5.920	3.289	2.631	6.925	6.409	5.562
66	1.058	15.812	16.471	24.097	5.930	3.294	2.635	6.938	6.420	5.572
67	1.058	15.837	16.497	24.132	5.939	3.299	2.640	6.950	6.432	5.581
68	1.059	15.862	16.523	24.167	5.948	3.305	2.644	6.962	6.443	5.591
69	1.060	15.887	16.549	24.202	5.958	3.310	2.648	6.974	6.454	5.601
70	1.061	15.912	16.575	24.237	5.967	3.315	2.652	6.987	6.466	5.611
71	1.062	15.937	16.601	24.272	5.976	3.320	2.656	6.999	6.477	5.621
72	1.063	15.962	16.627	24.307	5.986	3.325	2.660	7.011	6.489	5.631
73	1.064	15.987	16.653	24.342	5.995	3.331	2.664	7.024	6.500	5.641
74	1.065	16.012	16.679	24.377	6.004	3.336	2.669	7.036	6.511	5.650
75	1.066	16.037	16.705	24.412	6.014	3.341	2.673	7.048	6.523	5.660
76	1.067	16.062	16.731	24.447	6.023	3.346	2.677	7.061	6.534	5.670
77	1.068	16.087	16.757	24.482	6.033	3.351	2.681	7.073	6.546	5.680
78	1.069	16.112	16.783	24.517	6.042	3.357	2.685	7.085	6.557	5.690
79	1.070	16.137	16.809	24.552	6.051	3.362	2.689	7.098	6.569	5.700
80	1.071	16.162	16.835	24.587	6.061	3.367	2.694	7.110	6.580	5.710

**Table 4:** Monte Carlo simulation results.

Macrodamage model	Mean	SD	Variance	Probability of failure
The proposed femoral model using Miner's rule	0.333299	0.393134	0.154554	13.26%
Zioupos and Casinos [26]	0.806667	0.944787	0.892622	37.90%
Pattin et al. [25]	0.55734	0.246868	0.060944	42.20%
Hambli [13]	0.171942	0.393134	0.01129	16.63%

**Table 5:** The equations of the proposed models.

The models	The equations	*R* ^2^
The cortical bone model	*A* = −20.13*B*^5^ + 14.60*B*^4^ + 7.315*B*^3^–13.38B^2^ + 5.485*B*	0.973
The composite bone model	*A* = 3.706*B*^3^–5.202*B*^2^ + 2.402*B* + 0.009	0.982
The trabecular bone model	*A* = −31.84*B*^4^ + 31.52*B*^3^–13.66*B*^2^ + 2.573*B*	0.992
